# DNA Damage and Oxidative Stress in Human Disease

**DOI:** 10.1155/2013/696104

**Published:** 2013-09-19

**Authors:** Sharbel Weidner Maluf, Norma Possa Marroni, Vanina D. Heuser, Daniel Prá

**Affiliations:** ^1^Medical Genetics Service, Hospital de Clínicas de Porto Alegre, Rua Ramiro Barcelos, 2350, 90035-903 Porto Alegre, RS, Brazil; ^2^PPG Ciências Farmacêiticas, Universidade Federal do Piauí, 64049-550 Terezina, PI, Brazil; ^3^Genetics Service, Hospital Universitário, Universidade Federal de Santa Catarina, 88040-900 Florianópolis, SC, Brazil; ^4^PPG BioSaúde, Universidade Luterana do Brasil, Avenida Farroupilha 8001, 92425-900 Canoas, RS, Brazil; ^5^PPG Ciências Biológicas: Fisiologia, Universidade Federal do Rio Grande do Sul, Rua Sarmento Leite, 500, 90010-170 Porto Alegre, RS, Brazil; ^6^Department of Pathology, University of Turku, 20520 Turku, Finland; ^7^PPG em Promoção da Saúde, Universidade de Santa Cruz do Sul (UNISC), Avenida Independência, 2293, 96815-900 Santa Cruz do Sul, RS, Brazil

The impact of DNA damage in human diseases is gaining attention since the mid-1990s. DNA damage and oxidative stress are known factors for the origin and progression of cancer. DNA damage and oxidative stress have also been implicated in so diverse diseases such as brain injury, pulmonary diseases, and other chronic inflammation-related disorders.

This special issue contains eight papers, covering several aspects of the implication of DNA damage and oxidative stress. Three papers focus on brain, colorectal, and skin cancers and one paper focuses on the genomic stability and oxidative stress in the cancer-predisposing genetic syndrome ataxia telangiectasia. One paper discusses the implication of nutrients in genomic stability in cell cultures. Two papers discuss the effect of a vitamin and a neuroprotectant on diabetes and traumatic brain injury, respectively. Another paper focused on the impact of autophagy on idiopathic pulmonary fibrosis.

“*The vitamin D receptor (VDR) gene polymorphisms in Turkish brain cancer patients*” by B. Toptas et al. provides evidence for the first time that the risk of meningiomas might be related to polymorphisms in the nuclear receptor of vitamin D, an important factor for the regulation of cell division and proliferation. In “*Oxidative stress in the pathogenesis of colorectal cancer: cause or consequence?*” M. Per$\overset{ˇ}{\text{s}}$e reviews the interplay between the several risk factors that have been implicated in colorectal cancer, a very common type of cancer in Western countries, which has a complex etiology. N. C. Jenkins and D. Grossman show, in “*Role of melanin in melanocyte dysregulation of reactive oxygen species,*” that the presence of melanin in the skin appears to be a double-edged sword: it protects melanocytes as well as neighboring keratinocytes in the skin through its capacity to absorb UV radiation, but its synthesis in melanocytes results in higher levels of intracellular ROS that may increase melanoma susceptibility.

L. B. Ludwig et al. provide that ionizing radiation is more efficient than bleomycin to induce chromosomal instability in ataxia telangiectasia patients and that this instability is not related to a systemic increase in oxidative stress. In “*The influence of micronutrients in cell culture: a reflection on viability and genomic stability,*” A. L. V. Arigony et al. addresse the effect of several vitamins and minerals by reviewing their role in metabolic routes related to DNA homeostasis. The paper presents lines of evidence whether while in deficiency or excess in cell culture the micronutrients reviewed can reduce or increase the level of DNA damage and influence cell proliferation and viability. Finally, the authors advocate which nutrients should deserve more attention in future studies focusing on the increase of genomic stability and cell fitness under culture conditions.

“*Vitamin C intake reduces the cytotoxicity associated with hyperglycemia in prediabetes and type 2 diabetes*” by S. I. R. Franke et al. compares the levels of vitamin C intake, which is among the most abundant antioxidants obtained from diet, with the levels of markers of hyperglycemia, DNA damage, and cytotoxicity in subjects with type 2 diabetic or with risk of developing the disease. The authors observe that vitamin C intake slightly higher than the dietary recommendation for healthy individuals can be beneficial to the subjects by preventing the cell death of white blood cells that have been reported in the literature to be associated with diabetes complications.

In “*Therapeutic time window for edaravone treatment of traumatic brain injury in mice,*” K. Miyamoto et al. deal with the edaravone administration postcontrolled cortical impact (CCT) resulting in a significant reduction in the injury volume and oxidative stress. These findings suggest that edaravone could prove clinically useful to ameliorate the devastating effects of traumatic brain injury (TBI). “*Self-eating: friend or foe? The emerging role of autophagy in idiopathic pulmonary fibrosis,*” by G. A. Margaritopoulos et al. highlights some key issues regarding the process of autophagy and its possible association with the pathogenesis of idiopathic pulmonary fibrosis.

Advances in molecular biology and bioinformatics are allowing researchers to gain an increased understanding of the function and regulation of genes and to identify pathways that are affected. Currently, the search for biomarkers related to disease is gaining increasing attention and especially biomarkers for oxidative stress and DNA damage became more and more valuable instruments for unraveling disease pathogenesis and facilitating prediction, prevention, and treatment of diseases.

